# The Role of Inflammasome NLPR3 in the Development and Therapy of Periodontitis

**DOI:** 10.7150/ijms.74575

**Published:** 2022-09-21

**Authors:** Ying Zhao, Yue Quan, Ting Lei, Liumeizi Fan, Xin Ge, Sheng Hu

**Affiliations:** 1 Department of Anesthesiology & Perioperative Medicine, Xi'an People's Hospital (Xi'an Fourth Hospital), Xi'an, Shaanxi 710100, China.; 2 Department of Stomatology, Xi'an People's Hospital (Xi'an Fourth Hospital), Xi'an, Shaanxi 710100, China.

**Keywords:** Periodontitis, NLPR3 Inflammasome, Periodontal Ligament Cell, Osteoclast, Osteoblast, Leukocyte

## Abstract

Periodontitis is a chronic inflammatory disease that affects tooth-supporting tissues and even leads to tooth loss. NLRP3 inflammasomes play a critical role in periodontitis pathogenesis. Aberrant activation or overexpression of NLRP3 inflammasomes in cellular players, including osteoclasts, osteoblasts, periodontal ligament fibroblasts, and leukocytes often contributes to cellular dysfunction and environment abnormality, thus resulting in the disorganization of ligament and alveolar bone. In this review, we mainly focus on the negative regulation of NLRP3 inflammasome in periodontitis and highlight the importance of NLRP3 inflammasome as a candidate therapeutic target in periodontitis treatment. Then we elucidate the development status of NLRP3 inflammasome inhibitors and show their application potential for treating periodontitis. In summary, this review reveals the recent progress and perspectives of NLRP3 inflammasome and the therapeutic potential of NLRP3 inflammasome inhibitors in periodontitis.

## Introduction

Periodontitis is a chronic inflammatory condition caused by plaque-associated bacteria that cause an inflammatory reaction in the tooth supporting tissues. Periodontitis is the sixth most common disease in the world, affecting around 743 million people and having a high prevalence of 11.2 percent 1, 2. Periodontitis has become the most common cause of tooth loss all over the world 3. In addition, growing data suggests that periodontitis may be a risk factor for a variety of systemic diseases, such as cardiovascular diseases (CVD) 4, diabetes 5, Alzheimer's disease 6, rheumatoid arthritis 7, adverse pregnancy outcomes 8, and cancer 9. Periodontitis is not just a localized oral disease but also influences systemic health of individuals. Therefore, there is an urgent need to understand the mechanisms of periodontitis pathogenesis, which is essential for developing effective therapies and preventive approaches against periodontitis 1.

**Figure 1:**
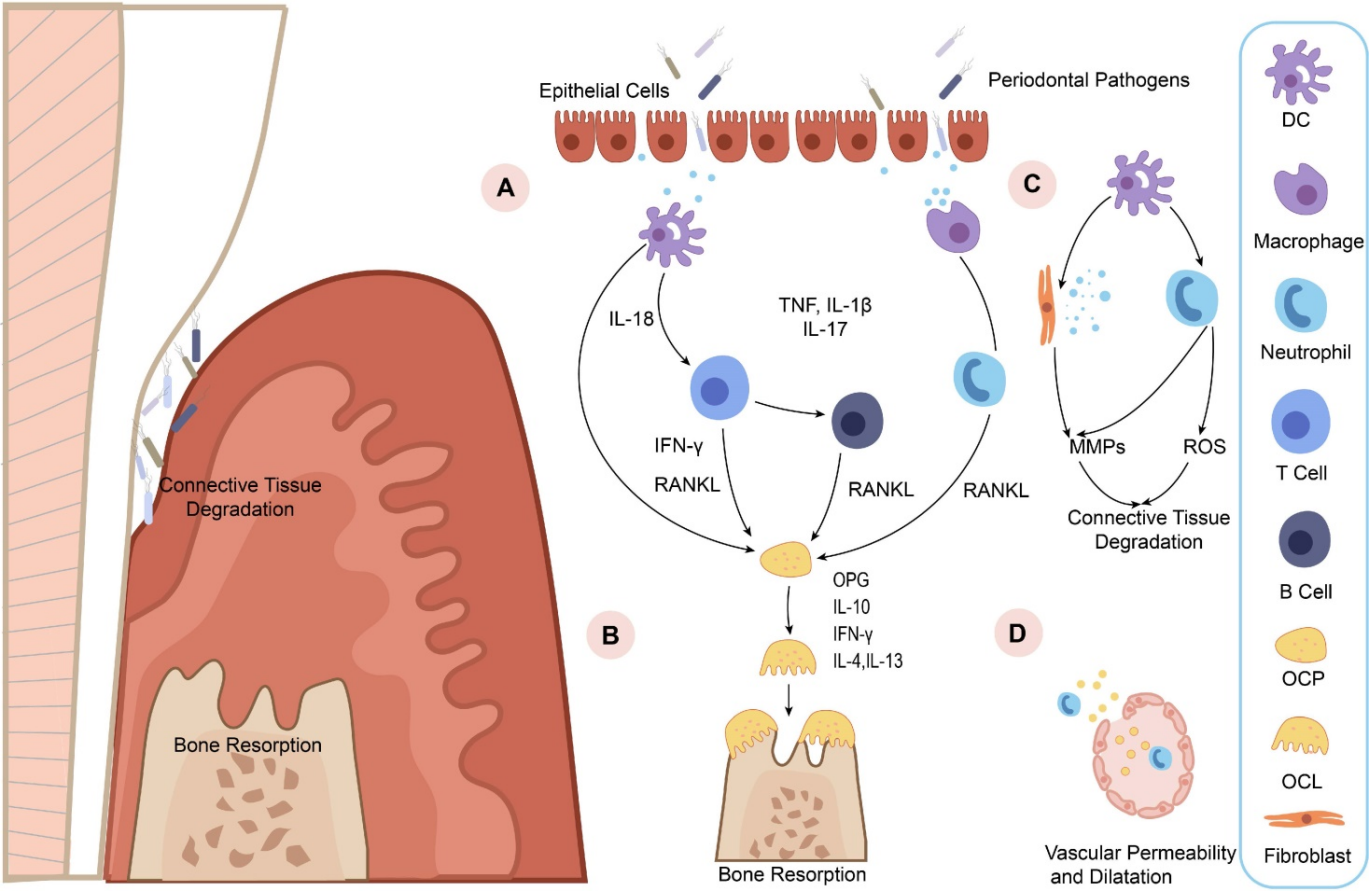
**Diagrammatic representation of periodontitis pathogenesis. A.** Increasing epithelial permeability leads to the invasion of pathogens, triggering immune cells to detect lipopolysaccharide (LPS) in the pathogens and pro-inflammatory cytokine production. Then the pro-inflammatory cytokines, such as tumor necrosis factor (TNF), interleukin-1β (IL-1β), interleukin-17 (IL-17), and IL-18 may activate neutrophils and osteoblasts to express RANKL and drive osteoclast maturation. **B.** OPG is an osteoclastogenesis inhibitor that acts as a soluble RANKL decoy receptor under inflammatory microenvironment. The imbalance of RANKL and OPG directly stimulates osteoclastogenesis. IL-10, IFN-γ, IL-4, and IL-13 then block osteoporosis by inhibiting osteoclastogenesis. **C.** Immune cells release MMPs and reactive oxygen species (ROS) to destruct and disorganize the extracellular matrix (ECM) in the periodontal tissue. **D.** Increased vascular permeability allows pro-inflammatory mediators and antimicrobial peptides to enter the bloodstream and causes inflammation in distal areas.

A great deal of evidence shows that both environmental and genetic factors contribute to periodontitis pathogenesis 10-12. Plaque biofilm is the initiating factor of periodontitis 13-15. The pathogenesis of periodontitis involves the complex interaction of multiple cell types, such as epithelial cells 16-18, immune cells 19-21, osteoclasts, osteoblasts, and periodontal ligament fibroblasts. It is worth noting that several activated proinflammatory transcription factors 11, 22, 23, inflammatory cytokines 24, and tissue-destructive molecules 25, 26 provide a network of signals to regulate the intracellular signaling which are vital for the pathological changes of periodontitis tissues (Figure 1). But unfortunately, the definition and diagnostic criteria of periodontitis have not been unified. Thus, based on the changes of inflammation related proteins, there have been an increasing number of studies searching for periodontitis biomarkers 10, 27, 28.

In recent years, increasing evidences confirmed that a complex of nucleotide-binding oligomerization domain-like receptor (NLR) complexes named “inflammasome” functions in periodontium immune response 29, 30. Inflammasomes are the master regulators of the innate immune system in chronic diseases, and they take part in controlling and limiting invading microbes 31. Appropriate inflammasome-mediated inflammation and cell death are conductive to reversing the adverse effects to promote tissue regeneration. Conversely, overexpression and excessive activation of the inflammasome often leads to uncontrolled inflammation, cytokine storm, tissue damage, and autoinflammatory and autoimmune diseases 32, 33. Similar to other early inflammatory related protein of periodontitis, growing evidences have shown elevated inflammasome levels in saliva and serum in patients with periodontitis, which correlate positively with the severity of periodontitis 27, 34-37. Therefore, in this review, we mainly focus on the negative regulation of inflammasome and illustrate the importance of inflammasome as a potential therapeutic target in periodontitis therapy.

To date, several inflammasomes have been described. NLRP3, as the most studied inflammasome, is activated by the infected pathogens and releasing of endogenous danger signals and then drives pathological inflammation in periodontitis 38-40. In this review, we describe the recent progress and our current understanding of NLRP3 inflammasome pathogenesis in periodontitis. With the goal to provide information for future study and clinical practice, we focus on the molecular mechanisms that activate and regulate excessive NLRP3 inflammasome, then we explore NLRP3 inflammasome activation and its physiopathological consequences in periodontitis. Finally, we also discuss the recently identified NLRP3 inflammasome inhibitors to provide insights into therapeutic strategies for treating periodontitis mediated by NLRP3 inflammasome.

## Inflammasome in periodontitis

**Figure 2:**
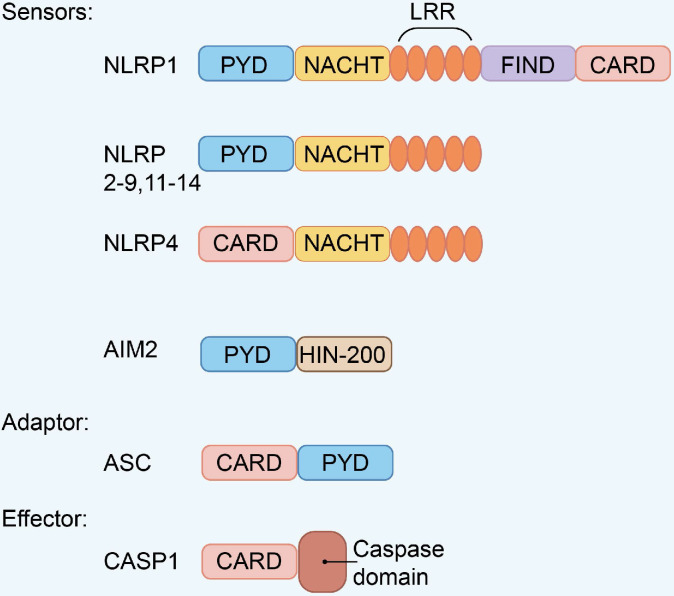
** Domain architecture of representative inflammasome**. Inflammasome family members with similar domain architectures including sensors, adaptor ASC, and effector CASP1.

Pattern recognition receptor (PRR) is related to the activation of host innate immune response and adaptive immunity to periodontal pathogens. Toll-like receptors (TLRs), nucleotide-binding oligomerization domain (NOD)-like receptors (NLRs), C-type lectin receptors (CLRs), and retinoic acid-inducible gene (RIG)-I-like receptors (RLRs) are members of the PRRs 41. PRRs can be activated in the host by recognizing molecules released by pathogens or damaged cells. These molecules are called pathogen-associated molecular patterns (PAMPs) and damage-associated molecular patterns (DAMPs) 42. The inflammasome formation requires the PRRs that play a crucial role in innate immunity. The etiology of numerous inflammatory illnesses, including periodontitis, is due to improper inflammasome activation. The inflammasome is a multi-protein complex, consisting of a PRR, an active form of caspase-1, and an adaptor protein or apoptosis-related speck-like protein containing a caspase activation and recruitment domain (CARD) (ASC) 43. Various types of inflammasomes have been identified, including Nod-like receptor pyrin domain-containing protein (NLRP1, NLRP2, NLRP3, NLRP6, NLRP12), NLR containing a CARD 4 (NLRC4), NLRC5, PYHINS, and absent in melanoma 2 (AIM2) 44. Structurally, these family members share similar domain architectures (Figure 2). Existing evidences show that periodontitis is connected to these inflammasomes.

NLRP1 is one of the first discovered inflammasomes, but how it is activated remains unclear, especially in periodontitis. NLRP1 contains CARD and pyrin domain (PYD) and mediates intracellular signaling processes including caspase-1 (CASP1) activation 45, 46. The expression level of NLRP1 inflammasome has been evaluated in chronic periodontitis (CP) and aggressive periodontitis (AgP) 47. Yilmaz et al. 48 reported that there was no difference in NLRP1 in *Porphyromonas gingivalis* (*P*.* gingivalis*) of human gingival epithelial cells. In AgP, NLRP1 showed a low expression level in the gingival tissues and expressed more frequently in the epithelium and connective tissues. These evidences suggest that the function of NLRP1 in periodontal disease remains unclear and needs further investigation. The AIM2 inflammasome has been reported in a variety of periodontitis investigations, and AIM2 has been identified as a susceptibility gene for periodontitis in a genome-wide association study (GWAS) with expression quantitative trait loci data 47, 49. AIM2 has been demonstrated to be expressed in periodontitis gingival tissue, including gingivitis, CP, and AgP 47, 50. The NLRP3 inflammasome is by far the best-studied and largest multimeric protein complex among these inflammasomes. The role of NLRP3 in periodontal disease has been extensively reported within the recent research. In what follows, we focus on the activation of NLRP3 inflammasome, the role of NLRP3 inflammasome in periodontitis pathogenesis and the therapeutic potential of NLRP3 inflammasome inhibitors in treating periodontitis.

## NLRP3 inflammasome

### The activation of NLRP3 inflammasome

**Figure 3:**
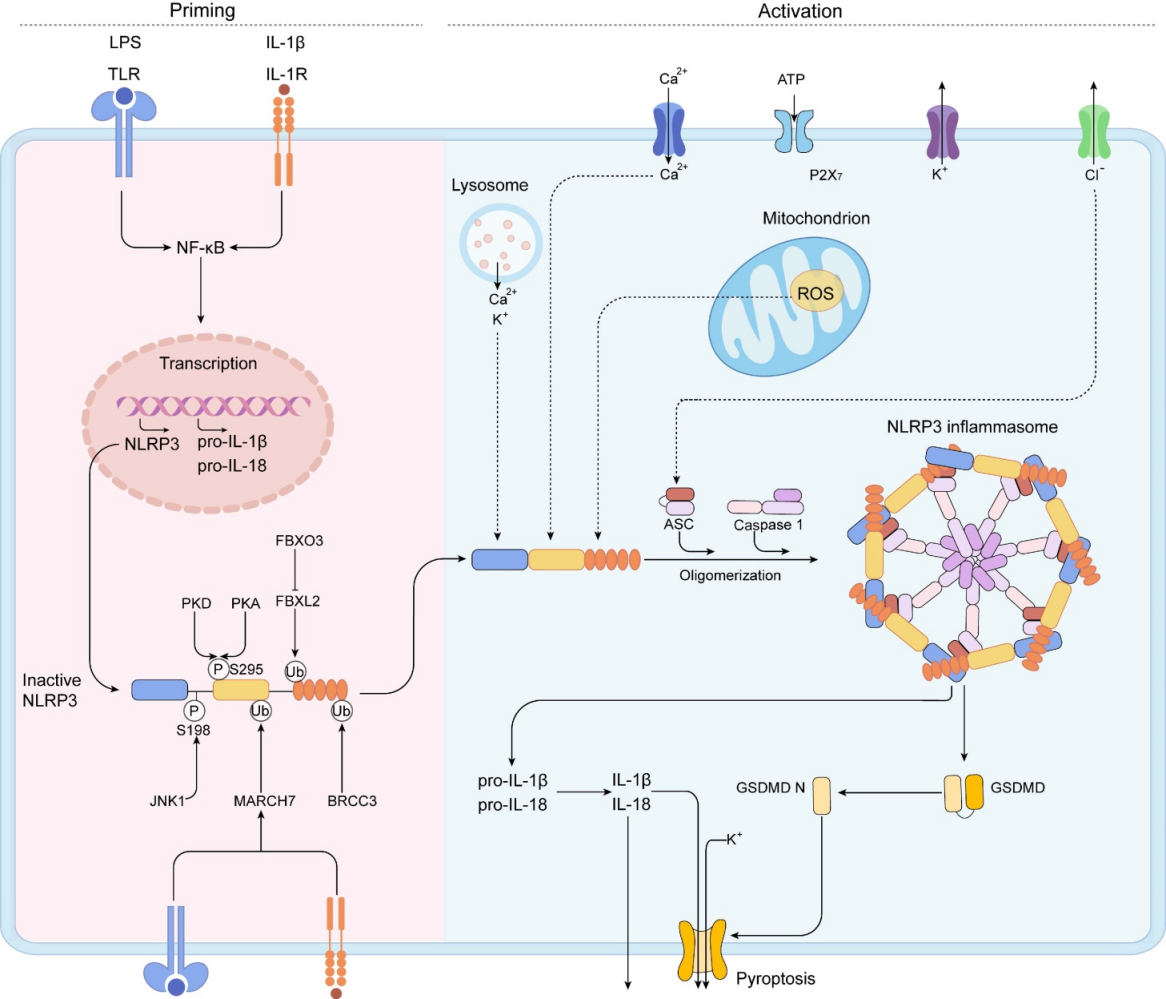
**A two-step mechanism of NLRP3 inflammasome activation**. The priming is triggered by the activation of cytokines or PAMPs, such as LPS and IL-1β, leading to the transcriptional upregulation of NLRP3 inflammasome components, including NLRP3, pro-IL-1β, pro-IL-18, caspase 1, and the activation of NF-κB signaling pathway. Post-translational modifications (PTMs) in the priming signal maintain NLRP3 in an auto-suppressed inactive conformation before stimulation. The self-oligomerization of NLRP3 occurs and the downstream recruitment of ASC is achieved by PYD-PYD interaction. Subsequently, aggregated ASC recruits pro-caspase-1, resulting in the activation of caspase-1. IL-1β, IL-18, and GSDMD can be activated by activated caspase-1. Then, caspase-1 cleaves GSDMD to liberate gasdermin D N-terminal form (GSDMD N). The GSDMD N can form membrane pores to mediate the nonconventional release of IL-1β and IL-18 and triggers pyroptosis.

NLRP3, a member of the NLR family of intracellular receptors, is a sensor that detects external pathogens and danger signals, triggering the formation and activation of the NLRP3 inflammasome. The adaptor (ASC) and effector (CASP1) are also contained in the NLRP3 inflammasome 51. The NLRP3 inflammasome is activated by two distinct signals: a priming signal and an activation signal (Figure 3) 52.

#### Priming step

Priming serves at least two steps. The first step is to upregulate NLRP3, pro-IL-1, and caspase 1 expression and activate the NF-B signaling cascades by recognizing PAMPs, DAMPs, or LPS that engage PRRs like TLRs.

The second step is to cause NLRP3 to undergo PTMs. PTMs stabilize NLRP3 in an auto-suppressed inactive conformation before stimulation. For NLRP3, diverse kinds of PTMs have been identified, including ubiquitylation, phosphorylation, and SUMO. The PYD domain of NLRP3 can be phosphorylated. NLRP3 activation is inhibited by phosphorylation at Ser3 53 and Tyr861 54, whereas phosphorylation of Ser198 by JUN N-terminal kinase 1 (JNK1) (also known as MAPK8) enhances NLRP3 activation 55. Protein kinase D (PKD) 56 phosphorylates NLRP3 to promotes NLRP3 activation, whereas PKA inhibits NLRP3 activation 57. Phosphorylation of Ser295 reduces NLRP3 ATPase activity and prevents NLRP3 activation 56. It is not clear why NLRP3 is phosphorylated at the same site by PKA and PKD but has the opposite effects 56, 58. Further studies are needed to clarify the phosphorylation of NLRP3.

By modulating the rate of NLRP3 breakdown, NLRP3 is deubiquitylated following priming and activation. NLRP3 Trp73 is recognized by Fbox/LRR-repeat protein 2 (FBXL2), which targets it for ubiquitylation and proteasomal destruction 59. TLR stimulation increases Fbox only protein 3 (FBXO3) expression, which degrades FBXL2. E3 ubiquitin ligase TRIM31 and membrane-associated RING finger protein 7 (MARCH7) triggered by TLR and IL1R activation cause Lys48-linked ubiquitylation and degradation of NLRP3 60, 61. The leucine-rich-repeat domain (LRR domain) of NLRP3 is deubiquitylated and homooligomerized by activated BRCC3 in response to priming signals 62. The protein E3 SUMO protein ligase MUL1 sumoylates NLRP3 in resting cells, inhibiting NLRP3 activation 63. SENP6 and SENP7 desumoylate NLRP3 after activation, promoting inflammasome activation 63. In conclusion, these results show how this dynamic landscape of PTMs delicately controls NLRP3 inflammasome activity. This crosstalk among the three different PTMs highlights the complex control of NLRP3 activation by the posttranslational regulation.

#### Activation step

When a primed cell is subjected to an activating stimulus, complete activation and the creation of an NLRP3 inflammasome ensue. The activation of the NLRP3 inflammasome is essential for caspase-1 autocatalytic activation. When NLRP3 is activated, it self-oligomerizes, allowing the downstream of ASC to be recruited via the PYD-PYD interaction. As a result of the CARD-CARD interaction, aggregated ASC attracts the effector, pro-caspase-1, culminating in caspase-1 activation 64, 65. The proteolytic activation of the proinflammatory cytokines IL-1β and IL-18, and of a pore forming protein, GSDMD, can be activated by activated caspase-1 heterotetramers through cleaving these substrates 66-68. After proteolysis, caspase-1 cleaves GSDMD to liberate GSDMD N. The oligomerized GSDMD N can generate membrane holes, allowing the nonconventional release of IL-1 and IL-18 and the induction of pyroptosis, a type of proinflammatory cell death. Multiple upstream cellular signals, including K^+^ or Cl^-^ efflux, Ca^2+^ flux, lysosomal disruption, mitochondrial dysfunction, metabolic alterations, and ROS generation, all contribute to NLRP3 inflammasome activation 65, 69. Despite the abundance of evidences defining the upstream signaling processes, no precise molecular events of NLRP3 activation have been identified yet.

### The role of NLRP3 inflammasome in periodontitis

NLRP3 and IL-1 are substantially expressed in human gingival tissues with severe chronic periodontitis 70, 71. IL-1β is essential for the pathogenesis and development of periodontitis, and NLRP3 inflammasome is engaged in the maturation of IL-1β and IL-18 72. The activation of NLRP3 inflammasomes has both beneficial and harmful impacts on the host's defensive system 73, 74. This section focuses on the role of NLRP3 inflammasomes in the pathogenesis and progression of periodontitis, provides an update on what is currently known about the effects of NLRP3 inflammasome activity on various cell types (including but not limited to osteoclasts, osteoblasts, gingival fibroblasts, periodontal ligament cells, and immune cells), and summarizes the current research on the potential role of NLRP3 inflammasomes in the treatment of periodontitis.

#### NLRP3 inflammasome and osteoclast

The result of periodontitis is the disorganization of ligament and alveolar bone 75. Studies on the pathogenesis of periodontitis have always focused on alveolar bone loss, especially the role of osteoclasts and osteoblasts during this process 76. A growing number of researches have revealed the critical role of osteoclasts and osteoblasts in the pathological changes of periodontitis 77-79.

Osteoclasts serve an important role in bone resorption during the process of periodontitis. The RANK/ receptor activator of NF-kB ligand (RANKL)/osteoprotegerin (OPG) axis is critical for osteoclastogenesis 80. The ability of RANKL to bind to receptor RANK upregulates nuclear factor of activated T cells 1 (NFATC1), a key transcription factor in osteoclast differentiation via recruiting TNF receptor-associated factor-6 (TRAF6) 81. Under physiological conditions, immature myeloid progenitors convert into multinucleated osteoclasts that correctly resorb bone tissue to ensure healthy bone turnover, whereas pathological states result in excessive bone loss 82. OPG is a RANKL receptor with a higher affinity than RANK, which inhibits osteoclastogenesis by binding to RANKL 81, 83. Some early inflammatory related proteins, such as transglutaminases, may regulate the alveolar bone loss by affecting the ratio of RANKL/OPG 28. Cytokines, particularly IL-1β and IL-18, processed by the effector caspase-1, may modulate osteoclast differentiation and activity either directly by effects on osteoclasts or indirectly by regulating RANKL expression by other cell types 84, 85. By upregulating the expression of cathepsin K and MMPs in periodontal tissues, IL-1β can boost the ability of osteoclasts to degrade extracellular matrix 86, 87.

Periodontal pathogens, such as *P*.* gingivalis*, can induce inflammatory responses associated with NLRP3 inflammasome signal transduction 88. Yohei et al. reported that the involvement of NLRP3 inflammasome was evaluated in *P*.* gingivalis*-induced periodontitis using NLRP3-KO mice 89. Infection-induced alveolar bone loss was significantly inhibited in NLRP3-KO mice, suggesting that NLRP3 inflammasome has mediated the production of inflammatory cytokines and has an important impact on *P*. gingivalis-induced bone loss 89. They also found significantly lower levels of RANKL and increased levels of OPG in NLRP3-KO mice, suggesting that NLRP3 inflammasomes may have functioned in promoting osteoclastogenesis in periodontitis mice 89. Kelk P et al. found that A. actinomycetemcomitans leukotoxicity mediates activation of the NLRP3 inflammasome in THP-1-differentiated macrophages which further facilitates osteoclasts differentiation 90. Another aging-related model of periodontitis also suggested that NLRP3 played an inevitable role in osteoclastogenesis during aging 91. An overactive NLRP3 inflammasome can boost osteoclast ability to resorb bone by rebuilding the actin cytoskeleton 92, autophagy, or ubiquitination 32. Therefore, by modulating osteoclast activity and differentiation, the NLRP3 inflammasome may be exploited as a target to treat periodontitis through governing bone resorption.

#### NLRP3 inflammasome and osteoblast

Collagen fibers, osteocalcin (OCN), and osteonectin are released by osteoblasts and function in bone deposition and mineralization 93. During osteoblast differentiation, other osteogenic markers such as alkaline phosphatase (ALP), runt-related transcription factor 2 (RUNX2), and osterix are also expressed. After the newly formed osteoid has calcified, osteoblasts evolve into osteocytes 93, 94. Then, osteocytes, osteoblasts, and osteoclasts form a network that demonstrates bone turnover 95. Osteoblasts express the core protein of inflammasome NLRP3 96. Infection of osteoblastic cells with pathogens results in the generation of IL-1β and IL-18, as well as apoptosis, which is mediated by the activation of the NLRP3 inflammasome 97, 98. These may affect the inflammatory bone resorption and bone turnover. Osteoblasts enhance osteoclastogenesis by increasing RANKL synthesis or decreasing OPG levels when the NLRP3 inflammasome is activated 99. In osteoblasts, chemokines induced by IL-1 govern osteoclast precursor migration and differentiation 100.

Study has indicated that multiple pathogens associated with periodontitis can cause NLRP3 inflammasome activation and apoptosis of osteoblast. A study has indicated that osteoblastic MG63 cells infected with periodontal bacteria *Aggregatibacter actinomycetemcomitans* promote apoptosis by activating the NLRP3 inflammasome 98. McCall et al. found that the apoptosis of osteoblasts after salmonella challenge requires functional expression of NLRP3 inflammasome 101.

Besides pathogenic bacteria, other factors have an impact on the NLRP3 inflammasome activation and osteogenic dysfunction. LPS treatment leads to the activation of NLRP3 inflammasome to mediate cell death, reduce cell migration and boost osteogenic dysfunction 102. ROS is a key factor in the NLRP3 inflammasome activation. Some studies have shown that the pathogenesis of periodontitis is related to ROS-induced oxidative stress 103, 104, which could accumulate in periodontal tissue and aggravate the damage to periodontal tissues. A study has shown that LPS-mediated increase of ROS elevates the NLRP3 inflammasome components IL-1β and IL-18, activates pyroptosis, and causes functional impairment in osteoblasts 105. These results suggest that ROS may promote alveolar bone loss in periodontitis by affecting the ROS-NLRP3-IL-1β pyroptosis axis in osteoblasts. It is possible to conclude that NLRP3 inflammasome activation lowers osteoblast activity by lowering its bone forming ability, differentiation, and proliferation, as well as triggering pyroptosis in osteoblasts and promoting bone resorption in periodontitis.

#### NLRP3 inflammasome and periodontal ligament fibroblasts

By producing cytokines and chemokines, human periodontal ligament fibroblasts (hPDLFs) contribute to periodontal inflammation, such as apical periodontitis and periodontitis 106. hPDLFs connect the teeth root to the alveolar bone and play an important role in repairing periodontal tissues and producing bone cells. Therefore, the therapeutic effect of periodontitis can be evaluated by cell proliferation, inflammation, and osteogenic induction ability of hPDLFs 107. Previous study suggested that NLRP3 and ASC were expressed stably in hPDLFs and mouse PDLFs 108. The activity of NLRP3 inflammasome induces periodontal inflammation and increasing proinflammatory cytokines such as IL-1β and IL-6, damaging the periodontal ligament 109. At present, there are relatively few studies on NLRP3 inflammasome in periodontal ligament fibroblasts, this is an issue warranting further research.

#### NLRP3 inflammasome and leukocytes

Previous study showed that fibroblasts and other stromal cells can specifically recruit leukocytes by expressing chemokines during the development of periodontitis 110. PAMP can be recognized by the host's PRRs on immune cells, leading to cell activation and production of cytokines and adhesion molecules 111. Neutrophils, also known as polymorphonuclear leukocytes, are the most abundant leukocytes in inflamed periodontal tissues and show a hyperactive state. The presence of severe periodontitis in patients with defective neutrophils displays the key role of neutrophils in periodontitis. The neutrophils infiltration in the area of periodontal lesion is dependent on NLRP3 expression 112. *Cheat et al.* reported that stimulation of neutrophils by *P. gingivalis* in WT mice increased NLRP3 and RANKL expression, activated osteoclasts and improved alveolar bone resorption. NLRP3 KO mice had almost no neutrophils in the gingival connective tissue, which may be responsible for the withdrawal of protective resorption of alveolar bone 33. These studies suggest that NLRP3 may be a switch that maintains/drives neutrophils in inflammatory tissues 112.

Macrophages play a critical role in the host defense system, and are involved in innate immune defense, activation of acquired immune response mediated by lymphocytes, initiation and resolution of inflammation, and alveolar bone resorption in periodontitis 113. Macrophages have two phenotypes, pro-inflammatory M1 and selective anti-inflammatory M2, which are determined by the microenvironment of surrounding tissues 114. Clinical studies have shown that the numbers of M1 and M2 macrophages in inflamed periodontal tissue were more than healthy tissue, of which M1 was dominant^115^, ^116^. The conversion from M2 to M1 macrophages is an important cause of periodontal tissue damage, the induction of M2 macrophage polarization may become a new alternative for treating periodontitis 117. These indicate the participation of NLRP3 inflammasome in periodontitis by regulating diverse types of leukocytes.

### Potential inhibitors of NLRP3 inflammasome in the therapy of periodontitis

The activation of NLRP3 inflammasome has been found in periodontal tissues of periodontitis patients. Negative regulation of the NLRP3 inflammasome is a potential therapeutic target for NLRP3-associated diseases. A number of NLRP3 inhibitors harbor the ability to inhibit NLRP3 inflammasome. And some of them have displayed their therapeutic potentials for treating periodontitis. However, the underlying mechanism or precise target is not fully understood. In the following part, we will discuss them in detail (Table 1).

#### MCC950

MCC950 (also referred to as CP-456,773) is a diarylsulfonylurea-containing compound that originally acts as an IL-1β inhibitor. Further study confirmed that MCC950 could directly interacts with the Walker B motif within the NLRP3 NACHT domain, and then blocking ATP hydrolysis and inhibiting NLRP3 activation and inflammasome formation. MCC950 has shown its therapeutic effects on periodontitis. MCC950 can significantly decrease the number and inhibit osteoclast differentiation, which ultimately results in the reduction of alveolar bone loss in mice with periodontitis 71, 91, 118. It can also present a beneficial therapeutic effect on periodontitis in a ligature-induced periodontitis mouse model, and acts directly on osteoclast precursors, reducing osteoclast development and alveolar bone loss in periodontitis 89. MCC950 could rescue the inhibition of osteogenesis in hPDLCs from inflammatory root resorption 39. Besides, MCC950 is able to ameliorate osteoblast migration and restore the expression of osteogenic differentiation-related proteins, such as RUNX2 and ALP, through inhibiting the activity of NLRP3 inflammasome 105. In summary, MCC950 may serve as a promising new treatment alternative for periodontitis by blocking NLRP3 inflammation and rescuing alveolar bone loss.

#### Glyburide

Glyburide (also known as glibenclamide) is an orally active ATP-sensitive K+ channel (K_ATP_) inhibitor which can be used for the study of diabetes and obesity. Previous study showed that glyburide could block NLRP3 inflammasome activation, decrease the production of proinflammatory mediators (TNF-α, IL-1β, and reactive oxygen species), and suppress the accumulation of inflammatory cells 119. While in the study of periodontitis, glyburide can prevent NLRP3 inflammasome activation and decrease IL-1β release in periodontal pathogen-induced inflammation 118. And oral administration of glyburide can lessen the alveolar bone resorption and osteoclastogenesis caused by traumatic occlusion in a rat model 120. Likewise, Jiang M et al. demonstrated that glyburide could reverse inflammation and bone resorption in occlusal trauma with periodontitis 121. These results suggest that glyburide application may achieve good treatment outcomes in periodontal therapy.

#### Tranilast

Tranilast (N-(3',4'-dimethoxycinnamonyl) anthranilic acid) was developed as an anti-allergic medication. Then it was utilized as an anti-inflammatory agent to treat inflammation-related diseases for its ability to bind to NLRP3 NACHT domain to block NLRP3-NLRP3 and NLRP3-ASC interactions. It has been shown to be therapeutically effective, exerting anti-inflammatory and anti-oxidative effects. Tranilast usage in periodontitis can partially alleviate apical periodontitis 122. During regulating bone homeostasis, tranilast inhibited activation of nuclear factor-κB and reduced induction and nuclear translocation of nuclear factor of activated T cells, ultimately leading to the inhibition of osteoclastogenesis by RANKL signaling 123. The same effect of tranilast inhibiting osteoclastogenesis has also been confirmed in the arthritis study 124. At present, application research on tranilast in periodontitis treatment is rare, but it deserves further investigations.

#### Irisin

Irisin is a peptide hormone originated from the cleaved fibronectin type III domain containing protein 5 (FNDC5). It can serve as an NLRP3 inhibitor through inhibiting NLRP3 inflammasome formation and activation caused by lipopolysaccharides 125 . Previous study showed that irisin facilitated primary hPDLCs proliferation and promoted osteogenic via increasing extracellular matrix formation 126. Under P. gingivalis-triggered inflammation, irisin facilitates the osteogenic/cementogenic differentiation of hPDLCs partially through the p38 signaling pathway 127. Interestingly, compared with other inhibitors, irisin promoted osteogenesis without osteoclast differentiation suppression. This result suggests that irisin would likely play a crucial role in tiny alveolar bone defects in periodontitis.

#### Melatonin

Melatonin (N-acetyl-5-methoxy tryptamine) is a hormone produced from L-tryptophan present mostly in the pineal gland, which is stored and released by salivary glands. Melatonin exerts inhibitory function on NLRP3 inflammasome activation through inhibiting or activating several proteins and pathways. Melatonin levels are associated with the severity of periodontitis. A systematic review by Balaji TM et al. has shown an initial reduction in melatonin levels followed by elevation with worsening of periodontitis 128. The application of melatonin as a topical/systemic formulation to treat periodontitis was also reported by Balaji TM et al. Apart from the level and application of melatonin in periodontitis, melatonin is demonstrated to regulate immune response and prevent periodontal tissue from damage. A meta-analysis showed that melatonin supplementation (topical and systemic) in periodontitis patients improved key periodontal parameters including pocket depth and clinical attachment loss 129. Researches of melatonin on bone repair and regeneration showed that melatonin could promote new bone regeneration and increase the number of osteoblast-like cells 130. A systematic review and meta-analysis of melatonin adjuvant therapy for periodontitis illustrated that melatonin can significantly improve the periodontal status after non-surgical treatment for periodontitis, and the efficacy is correlated with drug dose 131. On the contrary, it is noteworthy that different conclusions were also drawn by other researchers. Konečná B et al. reported that melatonin treatment had no significant impact on periodontitis 132. The authors also analyzed the negative outcome and deduced that this may relate to the different study duration and different melatonin doses. Although numerous clinical studies on melatonin have been performed, the impact of melatonin on periodontitis and its precise molecular mechanisms remain to be elusive. Further studies are still needed.

#### Dioscin

Dioscin (also referred to as CCRIS 4123 or Collettiside III) is a natural steroid saponin isolated from several plants. Previously it was used an anti-cancer reagent against various kinds of tumor cell lines. Recent study showed its potential to be a NLRP3 inflammasome inhibitor. Yin W et al. investigated the therapeutic effect of dioscin on periapical periodontitis in mouse, the results demonstrated that dioscin inhibited NLRP3 inflammasome activation in mouse macrophages and promoted the osteogenesis of mouse pre-osteoblasts 38. In addition, other studies confirmed that dioscin could reduce excessive inflammation and promote macrophage polarization to M2 phenotype 133. As a new candidate drug, the perspectives of dioscin are promising and deserve further investigations.

#### Parthenolide

Parthenolide is a sesquiterpene lactone found in the plant Tanacetum parthenium. Parthenolide exhibits anti-inflammatory activity by inhibiting NF-κB and HDAC1. It can also work as an NLRP3 inflammasome inhibitor through inhibiting NLRP3 ATPase activity. Parthenolide has been used to treat inflammation and inflammation related diseases. A few researches focused on the effects of parthenolide on treating periodontitis. Zhang X et al. revealed the anti-inflammatory and anti-osteoclastogenic effects of parthenolide and demonstrated its great potential for application in bone regeneration in periodontitis patients 134, 135. Research also showed that parthenolide could inhibit osteoclast differentiation by down-regulation of RANKL-mediated osteoclastogenesis. Furthermore, a novel targeted nano-parthenolide molecule has recently been developed for the treatment of acute myeloid leukemia 136. Based on the above research, whether parthenolide can be harnessed to develop target-directed drugs to periodontitis remains to be determined.

#### Other inflammasome inhibitors

Except for the inflammasome inhibitors described above, more inflammasome inhibitors have been developed, such as NLRP3-IN-2^137^, JC124 138, Arglabin 139, Isoandrographolide 140, Carvedilol 141, and JC-171 142. Unfortunately, their roles in periodontitis have not been clarified. But these indeed provide us enough alternative drugs for periodontitis treatment.

## Conclusions and Perspectives

The overexpression and activation of the NLRP3 inflammasome has been linked to the development of periodontitis in recent years. NLRP3 inflammasome exerts different regulatory functions in cells of different types in PDL. NLRP3 inflammasome enhances osteoclastogenesis by increasing RANKL synthesis or decreasing OPG levels. At the same time, NLRP3 inflammasome promotes apoptosis of osteoblasts, elevates proinflammatory cytokines in periodontal ligament fibroblast, and controls the functions of immune cells. These indicate the great potential of NLRP3 inflammasome as a target for treating periodontitis. Numerous NLRP3 inflammasome inhibitors have been developed and shown broad potentials for the therapeutic treatment of periodontitis. Nevertheless, the therapeutic effects and side effects of NLRP3 inflammasome inhibitors on periodontitis are largely unknown, and the precise mechanisms of how NLRP3 inflammasome inhibitors influence periodontitis are far from full elucidation. Despite all this, revealing the role of NLRP3 inflammasome in periodontitis pathogenesis and developing safe and effective NLRP3 inflammasome inhibitors will greatly contribute to periodontitis treatment.

## Figures and Tables

**Table 1:** NLRP3 inflammasome inhibitors in the therapy of periodontitis

Agents	Alias	Inhibition mechanism	Benefits
MCC950	CP-456,773	Directly interact with the Walker B motif within the NLRP3 NACHT domain, target the NLRP3 ATP-hydrolysis motif for inflammasome inhibition	Reduce the alveolar bone loss in periodontitis, decrease the differentiation of osteoclasts 71, 91, 118, and restore the osteogenic differentiation-related proteins expression 105.
Glyburide	Glibenclamide	Active ATP-sensitive K+ channel (K_ATP_) inhibitor, block NLRP3 inflammasome activation	Prevent NLRP3 inflammasome activation and decrease IL-1β release in periodontal pathogen-induced inflammation (116), lessen the alveolar bone resorption and osteoclastogenesis 120, reverse inflammation 121
Tranilast	N-(3',4'-dimethoxycinnamonyl) anthranilic acid	Bind to NLRP3 NACHT domain to block NLRP3-NLRP3 and NLRP3-ASC interaction	Alleviate apical periodontitis 122 and inhibit osteoclastogenesis 123, 124.
Irisin	FNDC5	Resist oxidative stress, formation and activation of NLRP3 inflammasome caused by lipopolysaccharides	Increase primary hPDLCs proliferation, promote osteogenic 126 and facilitate the osteogenic/ cementogenic differentiation of hPDLCs^127^
Melatonin	N-acetyl-5-methoxy tryptamine	Inhibitory function on NLRP3 inflammasome activation through inhibiting or activating several proteins and pathways	Improve key periodontal parameters including pocket depth and clinical attachment loss 129, promote new bone regeneration and increase the number of osteoblast-like cells 130.
Dioscin	CCRIS 4123 Collettiside III	Inhibit NF-κB, MAPK signaling and NLRP3 inflammasome	Inhibit the activation of NLRP3 inflammasome in macrophages and promote the osteogenesis, reduce excessive inflammation and promote macrophage polarization to M2 phenotype^133^
Parthenolide		Inhibit NLRP3 ATPase activity	Anti-inflammatory and anti-osteoclastogenic 134, 135

## Abbreviations

AgP: aggressive periodontitis
AIM2: absent in melanoma 2
ALP: alkaline phosphatase
AP-1: activator protein 1
ASC: apoptosis-related speck-like protein containing a caspase activation and recruitment domain (CARD)
CARD: caspase activation and recruitment domain
CASP1: caspase-1
CLRs: C-type lectin receptors
CP: chronic periodontitis
CVD: cardiovascular disease
DAMPs: damage-associated molecular patterns
FBXL2: Fbox/LRR-repeat protein 2
FBXO3: Fbox only protein 3
FIIND: function-to-find domain
GSDMD: gasdermin D
GSDMD N: gasdermin D N-terminal form
GWAS: genome-wide association study
IL-17: interleukin-17
IL-1β: interleukin-1β
JNK1: JUN N-terminal kinase 1
LPS: lipopolysaccharide
LRR: leucine-rich-repeat domain
MARCH7: membrane-associated RING finger protein 7
MMPs: matrix metalloproteinases
NACHT: nucleotide-binding oligomerization domain
NFATc1: Recombinant Nuclear Factor Of Activated T-Cells Cytoplasmic 1
NF-κB: nuclear factor kappa B
NLRs: nucleotide-binding and oligomerization domain (NOD)-like receptors
NLRP1: Nod-like receptor pyrin domain-containing protein 1
OCN: osteocalcin
PAMP: pathogen-associated molecular pattern
PKD: protein kinase D
PRR: pattern recognition receptor
PTMs: post-translational modifications
PYD: pyrin domain
RANKL: receptor activator of NF-kB ligand
RIG: retinoic acid-inducible gene
ROS: reactive oxygen species
RUNX2: runt-related transcription factor 2
SENP6: sentrin-specific protease 6
SUMO: sumoylation
TLRs: Toll-like receptors
TNF: tumor necrosis factor
TRIM31: tripartite motif containing protein 31

## References

[1] Peres MA, Macpherson LMD, Weyant RJ, Daly B, Venturelli R, Mathur MR (2019). Oral diseases: a global public health challenge. Lancet.

[2] Kinane DF, Stathopoulou PG, Papapanou PN (2017). Periodontal diseases. Nat Rev Dis Primers.

[3] Acharya A, VanWormer JJ, Waring SC, Miller AW, Fuehrer JT, Nycz GR (2013). Regional epidemiologic assessment of prevalent periodontitis using an electronic health record system. Am J Epidemiol.

[4] Sanz M, Marco Del Castillo A, Jepsen S, Gonzalez-Juanatey JR, D'Aiuto F, Bouchard P (2020). Periodontitis and cardiovascular diseases: Consensus report. J Clin Periodontol.

[5] Genco RJ, Borgnakke WS (2020). Diabetes as a potential risk for periodontitis: association studies. Periodontol 2000.

[6] Kamer AR, Craig RG, Niederman R, Fortea J, de Leon MJ (2020). Periodontal disease as a possible cause for Alzheimer's disease. Periodontol 2000.

[7] Potempa J, Mydel P, Koziel J (2017). The case for periodontitis in the pathogenesis of rheumatoid arthritis. Nat Rev Rheumatol.

[8] Bobetsis YA, Graziani F, Gursoy M, Madianos PN (2020). Periodontal disease and adverse pregnancy outcomes. Periodontol 2000.

[9] Nwizu N, Wactawski-Wende J, Genco RJ (2020). Periodontal disease and cancer: Epidemiologic studies and possible mechanisms. Periodontol 2000.

[10] Matarese G, Isola G, Ramaglia L, Dalessandri D, Lucchese A, Alibrandi A (2016). Periodontal biotype: characteristic, prevalence and dimensions related to dental malocclusion. Minerva Stomatol.

[11] Bartold PM (2018). Lifestyle and periodontitis: The emergence of personalized periodontics. Periodontol 2000.

[12] Eke PI, Wei L, Borgnakke WS, Thornton-Evans G, Zhang X, Lu H (2016). Periodontitis prevalence in adults >/= 65 years of age, in the USA. Periodontol 2000.

[13] Nunez J, Vignoletti F, Caffesse RG, Sanz M (2019). Cellular therapy in periodontal regeneration. Periodontol 2000.

[14] Carvalho CV, Saraiva L, Bauer FPF, Kimura RY, Souto MLS, Bernardo CC (2018). Orthodontic treatment in patients with aggressive periodontitis. Am J Orthod Dentofacial Orthop.

[15] Kwon T, Lamster IB, Levin L (2021). Current Concepts in the Management of Periodontitis. Int Dent J.

[16] Bosshardt DD (2018). The periodontal pocket: pathogenesis, histopathology and consequences. Periodontol 2000.

[17] Mysak J, Podzimek S, Sommerova P, Lyuya-Mi Y, Bartova J, Janatova T (2014). Porphyromonas gingivalis: major periodontopathic pathogen overview. J Immunol Res.

[18] Tadin A, Gavic L, Roguljic M, Jerkovic D, Zeljezic D (2019). Nuclear morphological changes in gingival epithelial cells of patients with periodontitis. Clin Oral Investig.

[19] Song L, Dong G, Guo L, Graves DT (2018). The function of dendritic cells in modulating the host response. Mol Oral Microbiol.

[20] Cury PR, Furuse C, Rodrigues AE, Barbuto JA, Araujo VC, Araujo NS (2008). Interstitial and Langerhans' dendritic cells in chronic periodontitis and gingivitis. Braz Oral Res.

[21] Ge X, Liu YF, Wong Y, Wu LZ, Tan L, Liu F (2016). Impact of nicotine on the interplay between human periodontal ligament cells and CD4+ T cells. Hum Exp Toxicol.

[22] Cavalla F, Osorio C, Paredes R, Valenzuela MA, Garcia-Sesnich J, Sorsa T (2015). Matrix metalloproteinases regulate extracellular levels of SDF-1/CXCL12, IL-6 and VEGF in hydrogen peroxide-stimulated human periodontal ligament fibroblasts. Cytokine.

[23] Wu LZ, Duan DM, Liu YF, Ge X, Zhou ZF, Wang XJ (2013). Nicotine favors osteoclastogenesis in human periodontal ligament cells co-cultured with CD4(+) T cells by upregulating IL-1beta. Int J Mol Med.

[24] Kolaczkowska E, Kubes P (2013). Neutrophil recruitment and function in health and inflammation. Nat Rev Immunol.

[25] Checchi V, Maravic T, Bellini P, Generali L, Consolo U, Breschi L (2020). The Role of Matrix Metalloproteinases in Periodontal Disease. Int J Environ Res Public Health.

[26] Sorsa T, Tjaderhane L, Salo T (2004). Matrix metalloproteinases (MMPs) in oral diseases. Oral Dis.

[27] Isola G, Polizzi A, Santonocito S, Alibrandi A, Williams RC (2022). Periodontitis activates the NLRP3 inflammasome in serum and saliva. J Periodontol.

[28] Matarese G, Curro M, Isola G, Caccamo D, Vecchio M, Giunta ML (2015). Transglutaminase 2 up-regulation is associated with RANKL/OPG pathway in cultured HPDL cells and THP-1-differentiated macrophages. Amino Acids.

[29] Rathinam VA, Fitzgerald KA (2016). Inflammasome Complexes: Emerging Mechanisms and Effector Functions. Cell.

[30] Man SM, Kanneganti TD (2015). Regulation of inflammasome activation. Immunol Rev.

[31] Owona BA, Abia WA, Moundipa PF (2020). Natural compounds flavonoids as modulators of inflammasomes in chronic diseases. Int Immunopharmacol.

[32] Murakami T, Nakaminami Y, Takahata Y, Hata K, Nishimura R (2022). Activation and Function of NLRP3 Inflammasome in Bone and Joint-Related Diseases. Int J Mol Sci.

[33] Cheat B, Torrens C, Foda A, Baroukh B, Sadoine J, Slimani L (2022). NLRP3 Is Involved in Neutrophil Mobilization in Experimental Periodontitis. Front Immunol.

[34] Isaza-Guzman DM, Medina-Piedrahita VM, Gutierrez-Henao C, Tobon-Arroyave SI (2017). Salivary Levels of NLRP3 Inflammasome-Related Proteins as Potential Biomarkers of Periodontal Clinical Status. J Periodontol.

[35] Altingoz SM, Kurgan S, Onder C, Serdar MA, Unluturk U, Uyanik M (2021). Salivary and serum oxidative stress biomarkers and advanced glycation end products in periodontitis patients with or without diabetes: A cross-sectional study. J Periodontol.

[36] Garcia-Hernandez AL, Munoz-Saavedra AE, Gonzalez-Alva P, Moreno-Fierros L, Llamosas-Hernandez FE, Cifuentes-Mendiola SE (2019). Upregulation of proteins of the NLRP3 inflammasome in patients with periodontitis and uncontrolled type 2 diabetes. Oral Dis.

[37] Belibasakis GN, Johansson A (2012). Aggregatibacter actinomycetemcomitans targets NLRP3 and NLRP6 inflammasome expression in human mononuclear leukocytes. Cytokine.

[38] Yin W, Liu S, Dong M, Liu Q, Shi C, Bai H (2020). A New NLRP3 Inflammasome Inhibitor, Dioscin, Promotes Osteogenesis. Small.

[39] Zhang J, Liu X, Wan C, Liu Y, Wang Y, Meng C (2020). NLRP3 inflammasome mediates M1 macrophage polarization and IL-1beta production in inflammatory root resorption. J Clin Periodontol.

[40] Olsen I, Yilmaz O (2016). Modulation of inflammasome activity by Porphyromonas gingivalis in periodontitis and associated systemic diseases. J Oral Microbiol.

[41] Guo H, Callaway JB, Ting JP (2015). Inflammasomes: mechanism of action, role in disease, and therapeutics. Nat Med.

[42] Lamkanfi M, Dixit VM (2014). Mechanisms and functions of inflammasomes. Cell.

[43] Strowig T, Henao-Mejia J, Elinav E, Flavell R (2012). Inflammasomes in health and disease. Nature.

[44] Xue Y, Enosi Tuipulotu D, Tan WH, Kay C, Man SM (2019). Emerging Activators and Regulators of Inflammasomes and Pyroptosis. Trends Immunol.

[45] Boyden ED, Dietrich WF (2006). Nalp1b controls mouse macrophage susceptibility to anthrax lethal toxin. Nat Genet.

[46] Taabazuing CY, Griswold AR, Bachovchin DA (2020). The NLRP1 and CARD8 inflammasomes. Immunol Rev.

[47] Xue F, Shu R, Xie Y (2015). The expression of NLRP3, NLRP1 and AIM2 in the gingival tissue of periodontitis patients: RT-PCR study and immunohistochemistry. Arch Oral Biol.

[48] Yilmaz O, Sater AA, Yao L, Koutouzis T, Pettengill M, Ojcius DM (2010). ATP-dependent activation of an inflammasome in primary gingival epithelial cells infected by Porphyromonas gingivalis. Cell Microbiol.

[49] Wang L, Sun L, Byrd KM, Ko CC, Zhao Z, Fang J (2020). AIM2 Inflammasome's First Decade of Discovery: Focus on Oral Diseases. Front Immunol.

[50] Aral K, Berdeli E, Cooper PR, Milward MR, Kapila Y, Karadede Unal B (2020). Differential expression of inflammasome regulatory transcripts in periodontal disease. J Periodontol.

[51] Agostini L, Martinon F, Burns K, McDermott MF, Hawkins PN, Tschopp J (2004). NALP3 forms an IL-1beta-processing inflammasome with increased activity in Muckle-Wells autoinflammatory disorder. Immunity.

[52] Denes BJ, Ait-Lounis A, Wehrle-Haller B, Kiliaridis S (2020). Core Matrisome Protein Signature During Periodontal Ligament Maturation From Pre-occlusal Eruption to Occlusal Function. Front Physiol.

[53] Stutz A, Kolbe CC, Stahl R, Horvath GL, Franklin BS, van Ray O (2017). NLRP3 inflammasome assembly is regulated by phosphorylation of the pyrin domain. J Exp Med.

[54] Spalinger MR, Kasper S, Gottier C, Lang S, Atrott K, Vavricka SR (2016). NLRP3 tyrosine phosphorylation is controlled by protein tyrosine phosphatase PTPN22. J Clin Invest.

[55] Song N, Liu ZS, Xue W, Bai ZF, Wang QY, Dai J (2017). NLRP3 Phosphorylation Is an Essential Priming Event for Inflammasome Activation. Mol Cell.

[56] Zhang Z, Meszaros G, He WT, Xu Y, de Fatima Magliarelli H, Mailly L (2017). Protein kinase D at the Golgi controls NLRP3 inflammasome activation. J Exp Med.

[57] Mortimer L, Moreau F, MacDonald JA, Chadee K (2016). NLRP3 inflammasome inhibition is disrupted in a group of auto-inflammatory disease CAPS mutations. Nat Immunol.

[58] Ye J, Zeng B, Zhong M, Li H, Xu L, Shu J (2021). Scutellarin inhibits caspase-11 activation and pyroptosis in macrophages via regulating PKA signaling. Acta Pharm Sin B.

[59] Han S, Lear TB, Jerome JA, Rajbhandari S, Snavely CA, Gulick DL (2015). Lipopolysaccharide Primes the NALP3 Inflammasome by Inhibiting Its Ubiquitination and Degradation Mediated by the SCFFBXL2 E3 Ligase. J Biol Chem.

[60] Yan Y, Jiang W, Liu L, Wang X, Ding C, Tian Z (2015). Dopamine controls systemic inflammation through inhibition of NLRP3 inflammasome. Cell.

[61] Song H, Liu B, Huai W, Yu Z, Wang W, Zhao J (2016). The E3 ubiquitin ligase TRIM31 attenuates NLRP3 inflammasome activation by promoting proteasomal degradation of NLRP3. Nat Commun.

[62] Py BF, Kim MS, Vakifahmetoglu-Norberg H, Yuan J (2013). Deubiquitination of NLRP3 by BRCC3 critically regulates inflammasome activity. Mol Cell.

[63] Barry R, John SW, Liccardi G, Tenev T, Jaco I, Chen CH (2018). SUMO-mediated regulation of NLRP3 modulates inflammasome activity. Nat Commun.

[64] Wang L, Hauenstein AV (2020). The NLRP3 inflammasome: Mechanism of action, role in disease and therapies. Mol Aspects Med.

[65] Bai B, Yang Y, Wang Q, Li M, Tian C, Liu Y (2020). NLRP3 inflammasome in endothelial dysfunction. Cell Death Dis.

[66] He WT, Wan H, Hu L, Chen P, Wang X, Huang Z (2015). Gasdermin D is an executor of pyroptosis and required for interleukin-1beta secretion. Cell Res.

[67] Ding J, Wang K, Liu W, She Y, Sun Q, Shi J (2016). Pore-forming activity and structural autoinhibition of the gasdermin family. Nature.

[68] Sharma BR, Kanneganti TD (2021). NLRP3 inflammasome in cancer and metabolic diseases. Nat Immunol.

[69] Yang Y, Wang H, Kouadir M, Song H, Shi F (2019). Recent advances in the mechanisms of NLRP3 inflammasome activation and its inhibitors. Cell Death Dis.

[70] Guan X, Guan Y, Shi C, Zhu X, He Y, Wei Z (2020). Estrogen deficiency aggravates apical periodontitis by regulating NLRP3/caspase-1/IL-1beta axis. Am J Transl Res.

[71] Zhong J, Pierantoni M, Weinkamer R, Brumfeld V, Zheng K, Chen J (2021). Microstructural heterogeneity of the collagenous network in the loaded and unloaded periodontal ligament and its biomechanical implications. J Struct Biol.

[72] Li Y, Ling J, Jiang Q (2021). Inflammasomes in Alveolar Bone Loss. Front Immunol.

[73] Xue Z, Zhang Z, Liu H, Li W, Guo X, Zhang Z (2019). lincRNA-Cox2 regulates NLRP3 inflammasome and autophagy mediated neuroinflammation. Cell Death Differ.

[74] Biasizzo M, Kopitar-Jerala N (2020). Interplay Between NLRP3 Inflammasome and Autophagy. Front Immunol.

[75] Huang X, Xie M, Xie Y, Mei F, Lu X, Li X (2020). The roles of osteocytes in alveolar bone destruction in periodontitis. J Transl Med.

[76] Yuan Y, Zhang H, Huang H (2021). microRNAs in inflammatory alveolar bone defect: A review. J Periodontal Res.

[77] Tsukasaki M (2021). RANKL and osteoimmunology in periodontitis. J Bone Miner Metab.

[78] Weng Y, Wang H, Li L, Feng Y, Xu S, Wang Z (2021). Trem2 mediated Syk-dependent ROS amplification is essential for osteoclastogenesis in periodontitis microenvironment. Redox Biol.

[79] Lu X, Zhu H, Chen Y, Wu Y, Zhang D, Zhu B (2021). A novel fluorescent probe for detecting hydrogen sulfide in osteoblasts during lipopolysaccharide-mediated inflammation under periodontitis. Sci Rep.

[80] Udagawa N, Koide M, Nakamura M, Nakamichi Y, Yamashita T, Uehara S (2021). Osteoclast differentiation by RANKL and OPG signaling pathways. J Bone Miner Metab.

[81] Ming J, Cronin SJF, Penninger JM (2020). Targeting the RANKL/RANK/OPG Axis for Cancer Therapy. Front Oncol.

[82] Kim JM, Lin C, Stavre Z, Greenblatt MB, Shim JH (2020). Osteoblast-Osteoclast Communication and Bone Homeostasis. Cells.

[83] Polzer K, Joosten L, Gasser J, Distler JH, Ruiz G, Baum W (2010). Interleukin-1 is essential for systemic inflammatory bone loss. Ann Rheum Dis.

[84] Kim JH, Jin HM, Kim K, Song I, Youn BU, Matsuo K (2009). The mechanism of osteoclast differentiation induced by IL-1. J Immunol.

[85] Jimi E, Nakamura I, Duong LT, Ikebe T, Takahashi N, Rodan GA (1999). Interleukin 1 induces multinucleation and bone-resorbing activity of osteoclasts in the absence of osteoblasts/stromal cells. Exp Cell Res.

[86] Heo SC, Kim YN, Choi Y, Joo JY, Hwang JJ, Bae MK (2021). Elevated Expression of Cathepsin K in Periodontal Ligament Fibroblast by Inflammatory Cytokines Accelerates Osteoclastogenesis via Paracrine Mechanism in Periodontal Disease. Int J Mol Sci.

[87] Behm C, Nemec M, Blufstein A, Schubert M, Rausch-Fan X, Andrukhov O (2021). Interleukin-1beta Induced Matrix Metalloproteinase Expression in Human Periodontal Ligament-Derived Mesenchymal Stromal Cells under In Vitro Simulated Static Orthodontic Forces. Int J Mol Sci.

[88] Wang X, Jia Y, Wen L, Mu W, Wu X, Liu T (2021). Porphyromonas gingivalis Promotes Colorectal Carcinoma by Activating the Hematopoietic NLRP3 Inflammasome. Cancer Res.

[89] Yamaguchi Y, Kurita-Ochiai T, Kobayashi R, Suzuki T, Ando T (2017). Regulation of the NLRP3 inflammasome in Porphyromonas gingivalis-accelerated periodontal disease. Inflamm Res.

[90] Kelk P, Moghbel NS, Hirschfeld J, Johansson A (2022). Aggregatibacter actinomycetemcomitans Leukotoxin Activates the NLRP3 Inflammasome and Cell-to-Cell Communication. Pathogens.

[91] Zang Y, Song JH, Oh SH, Kim JW, Lee MN, Piao X (2020). Targeting NLRP3 Inflammasome Reduces Age-Related Experimental Alveolar Bone Loss. J Dent Res.

[92] Qu C, Bonar SL, Hickman-Brecks CL, Abu-Amer S, McGeough MD, Pena CA (2015). NLRP3 mediates osteolysis through inflammation-dependent and -independent mechanisms. FASEB J.

[93] Hou YF, Shan C, Zhuang SY, Zhuang QQ, Ghosh A, Zhu KC (2021). Gut microbiota-derived propionate mediates the neuroprotective effect of osteocalcin in a mouse model of Parkinson's disease. Microbiome.

[94] Xu X, Zhang C, Trotter TN, Gowda PS, Lu Y, Ponnazhagan S (2020). Runx2 Deficiency in Osteoblasts Promotes Myeloma Progression by Altering the Bone Microenvironment at New Bone Sites. Cancer Res.

[95] Guder C, Gravius S, Burger C, Wirtz DC, Schildberg FA (2020). Osteoimmunology: A Current Update of the Interplay Between Bone and the Immune System. Front Immunol.

[96] Lei L, Sun J, Han J, Jiang X, Wang Z, Chen L (2021). Interleukin-17 induces pyroptosis in osteoblasts through the NLRP3 inflammasome pathway in vitro. Int Immunopharmacol.

[97] Ran S, Chu M, Gu S, Wang J, Liang J (2019). Enterococcus faecalis induces apoptosis and pyroptosis of human osteoblastic MG63 cells via the NLRP3 inflammasome. Int Endod J.

[98] Zhao P, Liu J, Pan C, Pan Y (2014). NLRP3 inflammasome is required for apoptosis of Aggregatibacter actinomycetemcomitans-infected human osteoblastic MG63 cells. Acta Histochem.

[99] Roper PM, Shao C, Veis DJ (2020). Multitasking by the OC Lineage during Bone Infection: Bone Resorption, Immune Modulation, and Microbial Niche. Cells.

[100] Matsuura T, Ichinose S, Akiyama M, Kasahara Y, Tachikawa N, Nakahama KI (2017). Involvement of CX3CL1 in the Migration of Osteoclast Precursors Across Osteoblast Layer Stimulated by Interleukin-1ss. J Cell Physiol.

[101] McCall SH, Sahraei M, Young AB, Worley CS, Duncan JA, Ting JP (2008). Osteoblasts express NLRP3, a nucleotide-binding domain and leucine-rich repeat region containing receptor implicated in bacterially induced cell death. J Bone Miner Res.

[102] Cao D, Pi J, Shan Y, Tang Y, Zhou P (2018). Anti-inflammatory effect of Resolvin D1 on LPS-treated MG-63 cells. Exp Ther Med.

[103] Sendur OF, Turan Y, Tastaban E, Serter M (2009). Antioxidant status in patients with osteoporosis: a controlled study. Joint Bone Spine.

[104] Sui BD, Xu TQ, Liu JW, Wei W, Zheng CX, Guo BL (2013). Understanding the role of mitochondria in the pathogenesis of chronic pain. Postgrad Med J.

[105] Liu S, Du J, Li D, Yang P, Kou Y, Li C (2020). Oxidative stress induced pyroptosis leads to osteogenic dysfunction of MG63 cells. J Mol Histol.

[106] Jiang N, Guo W, Chen M, Zheng Y, Zhou J, Kim SG (2016). Periodontal Ligament and Alveolar Bone in Health and Adaptation: Tooth Movement. Front Oral Biol.

[107] Uchiyama M, Nakamichi Y, Nakamura M, Kinugawa S, Yamada H, Udagawa N (2009). Dental pulp and periodontal ligament cells support osteoclastic differentiation. J Dent Res.

[108] Lu WL, Song DZ, Yue JL, Wang TT, Zhou XD, Zhang P (2017). NLRP3 inflammasome may regulate inflammatory response of human periodontal ligament fibroblasts in an apoptosis-associated speck-like protein containing a CARD (ASC)-dependent manner. Int Endod J.

[109] Lian D, Dai L, Xie Z, Zhou X, Liu X, Zhang Y (2018). Periodontal ligament fibroblasts migration injury via ROS/TXNIP/Nlrp3 inflammasome pathway with Porphyromonas gingivalis lipopolysaccharide. Mol Immunol.

[110] Williams DW, Greenwell-Wild T, Brenchley L, Dutzan N, Overmiller A, Sawaya AP (2021). Human oral mucosa cell atlas reveals a stromal-neutrophil axis regulating tissue immunity. Cell.

[111] Xu W, Zhou W, Wang H, Liang S (2020). Roles of Porphyromonas gingivalis and its virulence factors in periodontitis. Adv Protein Chem Struct Biol.

[112] Inoue Y, Shirasuna K, Kimura H, Usui F, Kawashima A, Karasawa T (2014). NLRP3 regulates neutrophil functions and contributes to hepatic ischemia-reperfusion injury independently of inflammasomes. J Immunol.

[113] Hou L, Ye Y, Gou H, Tang H, Zhou Y, Xu X (2022). A20 inhibits periodontal bone resorption and NLRP3-mediated M1 macrophage polarization. Exp Cell Res.

[114] Han Y, Huang Y, Gao P, Yang Q, Jia L, Zheng Y (2022). Leptin Aggravates Periodontitis by Promoting M1 Polarization via NLRP3. J Dent Res.

[115] Zhou LN, Bi CS, Gao LN, An Y, Chen F, Chen FM (2019). Macrophage polarization in human gingival tissue in response to periodontal disease. Oral Dis.

[116] Wu X, Chen H, Wang Y, Gu Y (2020). Akt2 Affects Periodontal Inflammation via Altering the M1/M2 Ratio. J Dent Res.

[117] Yu T, Zhao L, Huang X, Ma C, Wang Y, Zhang J (2016). Enhanced Activity of the Macrophage M1/M2 Phenotypes and Phenotypic Switch to M1 in Periodontal Infection. J Periodontol.

[118] Kawahara Y, Kaneko T, Yoshinaga Y, Arita Y, Nakamura K, Koga C (2020). Effects of Sulfonylureas on Periodontopathic Bacteria-Induced Inflammation. J Dent Res.

[119] Zhang G, Lin X, Zhang S, Xiu H, Pan C, Cui W (2017). A Protective Role of Glibenclamide in Inflammation-Associated Injury. Mediators Inflamm.

[120] Arita Y, Yoshinaga Y, Kaneko T, Kawahara Y, Nakamura K, Ohgi K (2020). Glyburide inhibits the bone resorption induced by traumatic occlusion in rats. J Periodontal Res.

[121] Jiang M, Shang Z, Zhang T, Yin X, Liang X, Sun H (2022). Study on the role of pyroptosis in bone resorption induced by occlusal trauma with or without periodontitis. J Periodontal Res.

[122] Kawakami T, Fukai K, Sowa J, Ishii M, Teramae H, Kanazawa K (2008). Case of cheilitis granulomatosa associated with apical periodontitis. J Dermatol.

[123] Phan TV, Ke K, Sul OJ, Park YK, Kim KK, Cho YS (2014). Protection against ovariectomy-induced bone loss by tranilast. PLoS One.

[124] Shiota N, Kovanen PT, Eklund KK, Shibata N, Shimoura K, Niibayashi T (2010). The anti-allergic compound tranilast attenuates inflammation and inhibits bone destruction in collagen-induced arthritis in mice. Br J Pharmacol.

[125] Deng X, Huang W, Peng J, Zhu TT, Sun XL, Zhou XY (2018). Irisin Alleviates Advanced Glycation End Products-Induced Inflammation and Endothelial Dysfunction via Inhibiting ROS-NLRP3 Inflammasome Signaling. Inflammation.

[126] Pullisaar H, Colaianni G, Lian AM, Vandevska-Radunovic V, Grano M, Reseland JE (2020). Irisin promotes growth, migration and matrix formation in human periodontal ligament cells. Arch Oral Biol.

[127] Huang X, Xiao J, Wang X, Cao Z (2022). Irisin attenuates P. gingivalis-suppressed osteogenic/cementogenic differentiation of periodontal ligament cells via p38 signaling pathway. Biochem Biophys Res Commun.

[128] Balaji TM, Varadarajan S, Jagannathan R, Gupta AA, Raj AT, Patil S (2022). Melatonin levels in periodontitis vs. the healthy state: A systematic review and meta-analysis. Oral Dis.

[129] Balaji TM, Varadarajan S, Jagannathan R, Mahendra J, Fageeh HI, Fageeh HN (2021). Melatonin as a Topical/Systemic Formulation for the Management of Periodontitis: A Systematic Review. Materials (Basel).

[130] Lu X, Yu S, Chen G, Zheng W, Peng J, Huang X (2021). Insight into the roles of melatonin in bone tissue and bonerelated diseases (Review). Int J Mol Med.

[131] Liu RY, Li L, Zhang ZT, Wu T, Lin S, Zhang XT (2022). Clinical efficacy of melatonin as adjunctive therapy to non-surgical treatment of periodontitis: a systematic review and meta-analysis. Inflammopharmacology.

[132] Konecna B, Chobodova P, Janko J, Banasova L, Babickova J, Celec P (2021). The Effect of Melatonin on Periodontitis. Int J Mol Sci.

[133] Cai J, Liu J, Fan P, Dong X, Zhu K, Liu X (2021). Dioscin prevents DSS-induced colitis in mice with enhancing intestinal barrier function and reducing colon inflammation. Int Immunopharmacol.

[134] Zhang X, Fan C, Xiao Y, Mao X (2014). Anti-inflammatory and antiosteoclastogenic activities of parthenolide on human periodontal ligament cells in vitro. Evid Based Complement Alternat Med.

[135] Zhang X, Chen Q, Liu J, Fan C, Wei Q, Chen Z (2017). Parthenolide Promotes Differentiation of Osteoblasts Through the Wnt/beta-Catenin Signaling Pathway in Inflammatory Environments. J Interferon Cytokine Res.

[136] Darwish NHE, Sudha T, Godugu K, Bharali DJ, Elbaz O, El-Ghaffar HAA (2019). Novel Targeted Nano-Parthenolide Molecule against NF-kB in Acute Myeloid Leukemia. Molecules.

[137] Zheng J, Jiang Z, Song Y, Huang S, Du Y, Yang X (2022). 3,4-Methylenedioxy-beta-Nitrostyrene Alleviates Dextran Sulfate Sodium-Induced Mouse Colitis by Inhibiting the NLRP3 Inflammasome. Front Pharmacol.

[138] Kuwar R, Rolfe A, Di L, Xu H, He L, Jiang Y (2019). A novel small molecular NLRP3 inflammasome inhibitor alleviates neuroinflammatory response following traumatic brain injury. J Neuroinflammation.

[139] Manayi A, Nabavi SM, Khayatkashani M, Habtemariam S, Khayat Kashani HR (2021). Arglabin could target inflammasome-induced ARDS and cytokine storm associated with COVID-19. Mol Biol Rep.

[140] Ahmed S, Kwatra M, Ranjan Panda S, Murty USN, Naidu VGM (2021). Andrographolide suppresses NLRP3 inflammasome activation in microglia through induction of parkin-mediated mitophagy in in-vitro and in-vivo models of Parkinson disease. Brain Behav Immun.

[141] Wong WT, Li LH, Rao YK, Yang SP, Cheng SM, Lin WY (2018). Repositioning of the beta-Blocker Carvedilol as a Novel Autophagy Inducer That Inhibits the NLRP3 Inflammasome. Front Immunol.

[142] Guo C, Fulp JW, Jiang Y, Li X, Chojnacki JE, Wu J (2017). Development and Characterization of a Hydroxyl-Sulfonamide Analogue, 5-Chloro-N-[2-(4-hydroxysulfamoyl-phenyl)-ethyl]-2-methoxy-benzamide, as a Novel NLRP3 Inflammasome Inhibitor for Potential Treatment of Multiple Sclerosis. ACS Chem Neurosci.

